# The Effects of the Ward Environment and Language in Palliative Care: A Qualitative Exploratory Study of Victorian Nurses’ Perspectives

**DOI:** 10.1177/19375867231177299

**Published:** 2023-06-02

**Authors:** Elizabeth M. Miller, Joanne E. Porter, Michael S. Barbagallo

**Affiliations:** 1 Collaborative Evaluation and Research Group (CERG), Federation University Australia, Churchill, Victoria, Australia; 2 Institute of Health and Wellbeing, Federation University Australia, Churchill, Victoria, Australia

**Keywords:** therapeutic landscapes, bad news, hospital environment, language, palliative, COVID-19, qualitative research, home-like, end-of-life, acute ward

## Abstract

**Objectives:**

The current study aimed to explore regional nurses’ perspectives of how bad news is delivered and the physical, natural, social, and symbolic environments where these conversations occur.

**Background:**

In regional hospitals within Victoria, Australia, palliative and end-of-life patients are cared for in acute wards that are often busy, noisy, and do not have a palliative psychosocial focus. On the other hand, Palliative Care Units (PCUs) have more home-like dedicated spaces, yet nearly all these facilities are in metropolitan areas. Diagnostic/prognostic (bad news) conversations about life-limiting illnesses often occur at the bedside in both environments.

**Method:**

Nurses providing palliative or end-of-life care in regional or metropolitan Victorian hospital inpatient wards were invited to interview and recruited through social media and snowballing. Six semi-structured, audio-recorded online interviews were conducted between March and May 2022, and themes were developed using reflexive thematic analysis.

**Results:**

Semi-structured online interviews were conducted with six female, registered nurses, four of whom worked in regional Victorian hospitals and two in metropolitan PCUs as Nurse Unit Managers. Three central themes were developed: “conducting family meetings,” “palliative care practice,” and “the environment matters.”

**Conclusions:**

A therapeutic environment for palliative patients and their families consists of home-like ambience and aesthetics and a psychosocial environment created by staff who can provide holistic palliative care. Holistic palliative care requires mentoring and mirroring of expert practice to increase the expertise and capacity of the palliative care workforce in acute general hospital wards.

## Introduction

Delivering bad news regarding a life-limiting diagnosis or providing an update that treatment is no longer working is a problematic aspect of a doctor’s role (Miller et al., 2022a), despite education, training, and the availability of multiple guidelines and protocols (Alelwani & Ahmed, 2014; Carrard et al., 2020; Sobczak, 2022; von Blanckenburg et al., 2020). Developing a therapeutic relationship between doctor and patient assists in delivering bad news (Berkey et al., 2018). However, the doctor’s culture, beliefs, confidence, and training level can hinder open and frank discussion (Miller et al., 2023).

Often bad news is delivered to the patient and family within an organized family meeting with other health professionals present (Cahill et al., 2016) and nurses, who spend the majority of time with patients and family members, often interpret and reinforce the diagnosis or prognosis (Glajchen & Goehring, 2017). Patients and family members rely on the information delivered to them by health professionals to plan their care and set goals toward achieving quality end-of-life (Kogan et al., 2013; Miller et al., 2022a; Rao et al., 2016). Therefore, the message must be delivered clearly and directly to avoid misunderstanding and confusion (Wentlandt et al., 2018).

Hospitalized palliative and end-of-life patients have different physical and psychosocial needs than other acute patients (Miller et al., 2022b) yet are often cared for in the same fast-paced, noisy, task-focused environment (Chan et al., 2018), especially in regional areas of Victoria, Australia, that do not have a Palliative Care Unit (PCU). In addition, COVID-19 has severely impacted the physical and psychosocial hospital experience for patients, family members, and staff (Bloomer & Walshe, 2021; Kirby et al., 2021; Klinger et al., 2022; Rokach, 2022; Usher et al., 2020), creating a nontherapeutic environment, contrary to the needs of palliative and end-of-life patients and their families. Nurses providing care to patients and their families create some of these environments and witness how they affect the patients they care for.

The study aimed to explore and understand the experiences and perceptions of registered nurses who provide palliative and end-of-life care in acute wards in regional hospitals and metropolitan PCUs in Victoria, Australia, to understand (1) bad news delivery and the language used when communicating with patients and family members; and (2) the built, natural, social, and symbolic environments where care is provided.

The experience and perceptions of nurses will help inform person-centered care, clinical practice, and training around the delivery of bad news and ward environmental factors and broaden the application of the Therapeutic Landscapes framework within palliative care (Gesler, 1992). When nurses share their experiences about providing palliative and end-of-life care, they help create the latest evidence to inform education, healthcare policy, and hospital design.

## Method

### Theoretical Framework

Gesler first coined Therapeutic Landscapes in 1992, which described four different environments (the built, natural, social, and symbolic) that, when positive and combined, an environment conducive to healing is developed. “Healing” is holistic, encompasses the mental, spiritual, and relational domains, and is especially relevant to palliative care (World Health Organisation [WHO], 2018). The Therapeutic Landscapes theoretical framework (Gesler, 1992; Gesler, 1996) was chosen to guide the study as little is known about how these four environments are represented within the inpatient palliative care areas, especially their impact on bad news delivery.

### Design

A qualitative exploratory design (Polit & Beck, 2017) was used for this study using many techniques described by case study theorists as the study forms part of a case study examining the delivery of bad news within the acute hospital environment. The exploratory approach aimed to explore and understand the participants’ lived experiences with the phenomenon in acute hospitals within Victoria, Australia.

### Population

English-speaking registered nurses who provide palliative or end-of-life care within acute/sub-acute Victorian hospital wards (excluding the intensive care unit and emergency departments) were invited to participate in an online semi-structured interview.

### Ethical Considerations

Due to the topic’s sensitive nature, the participant was asked to insert a support person’s name and contact number at the back of the consent form so the researcher could contact them if they became distressed during the interview. Federation University Australia granted ethical approval (A22-015) on February 23, 2022.

### Data Collection and Analysis

Participants were recruited through three advertisements on social media community pages across regional Victoria and four advertisements placed in nursing association e-newsletters between March and May 2022. Ten interested participants responded via social media platforms or email and were followed up and screened, resulting in three not meeting the inclusion criteria. After screening, seven were sent a Plain Language Information Statement and Consent Form, but only six progressed to an interview. Participants provided informed written consent prior to the commencement of their interview, which was conducted using a semi-structured interview technique, audio-recorded, and promptly transcribed verbatim. After each interview, the first author wrote their reflection and insights, which helped inform the following interviews, similar to a constant comparative method described by Merriam and Tisdell (2016). Early meaning-based themes were written in the margins of each transcript, making the authors confident that enough rich, thick data had been gathered to develop “meaning sufficiency” or “information power” (asserted by Braun & Clarke to be a more correct term than “data saturation”) to answer the research question and interviewing ceased (Braun & Clarke, 2022; Malterud et al., 2016). The three authors manually coded the de-identified data into patterns of meaning (Stake, 1995) using reflexive thematic analysis (Braun & Clarke, 2006, 2019).

### Trustworthiness and Reflexivity

To aid consistency and credibility, data were triangulated against themes from patient and family member experiences within a larger case study (Miller et al., 2023). The first author is a registered nurse undertaking her doctorate in palliative care and is supported by experienced researchers. The first author maintained a reflexive journal throughout the project and after each interview. In addition, weekly meetings and notes provide an audit trail.

## Results

Six individual, semi-structured interviews were conducted with participants between March and May 2022. Three were conducted via an online video platform, two in their work office, and one at home. Two interviews lasted 90 min, while four were completed in 60 min. Further details of participant demographics can be found in Table 1.

**Table 1.** Participant Demographics.

Participant	1	2	3	4	5	6
Role	RN	ANUM	NP	NUM	RN	NUM
Age (years)	35	39	56	66	53	45
Experience (years)	12	18	34	19	2	22
Postgrad education	None	None	Masters	Masters	None	Grad certificate
Region	Regional	Regional	Regional	Metro	Regional	Metro

*Note*. RN = registered nurse; ANUM = associate nurse unit manager; NP = nurse practitioner; NUM = nurse unit manager.

Data analysis resulted in three central themes and eight subthemes that explored bad news delivery and language, the role of registered nurses in providing palliative care in an acute hospital setting, and the impact that COVID-19 has had on their practice. The third theme explored nurses’ perceptions of their current and ward environments and described their ideal palliative environments. The central and subthemes are presented in Table 2. To protect the anonymity of the participants, quotes have not been labeled.

**Table 2.** Themes.

Conducting family meetings	Having hard conversations Ensuring understanding
Palliative care practice	Aiming for holistic patient care Palliative care training COVID-19 impact on practice
The environment matters	The clinical environment A home-like environment Connection to nature

## Conducting Family Meetings

This theme discusses the difficulty of using direct language when delivering bad news, the importance of family meetings, and the role registered nurses play in assisting patients and families in understanding what was discussed. The acute generalist nurses reported not delivering bad news themselves, whereas the Nurse Unit Managers (NUMs) in the PCUs did.

### Having Hard Conversations

Nurses reported that in their experience, “Death and dying is still a huge taboo in our society, that no one wants to talk about. Even down to nursing notes…to see ‘dying or death’ written is very rare.” The following example highlighted some ways nurses and doctors used avoidance strategies.

Their first approach when talking about death and dying is to deflect and to fluff and to use soft language and to avoid really, and resident doctors are the same. It’s not part of their training.

Soft language, used to soften a word’s literal meaning, occurred across the regional acute wards and the metropolitan PCUs. “A lot of the staff here use metaphors. And I say, ‘Come on, guys, we’re in a palliative care unit. We’re meant to be setting the examples here.’” Nurses reported this was not only due to a lack of training but “a lot of the time that’s cultural, they’re not used to it.” As a result, the tendency to avoid was often reflected in late referrals to the palliative care team. “I often find that some of the medical teams don’t refer patients, but the nurses have been asking for days, ‘You need to refer to palliative care!’”

***Death and dying is still a huge taboo in our society, that no one wants to talk about. Even down to nursing notes…to see ‘dying or death’ written is very rare.***

***A lot of the staff here use metaphors. And I say, ‘Come on, guys, we’re in a palliative care unit. We’re meant to be setting the examples here.’***

Family meetings were regarded as a “really good opportunity to bring everybody to hear the same message…It’s a chance to clarify and…for them to ask questions and have those questions addressed.” There was a difference in the content, difficulty, and complexity of family meetings between the regional acute wards and the PCU. Nurses reported that family meetings were often held at the bedside, but when that space was not suitable in the regional acute wards, often any room was used.

We don’t have a designated room for the family meetings, which we do really need as well, because that is very much a multipurpose room…It probably still feels like a very clinical environment… The walls are messy because it’s got all the pin-up boards and everything in there with staff education…but it would be nice to have a room that’s much more gentle, I suppose, that is set up to actually sit and just chat.

In contrast, both PCUs had nonclinical spaces with a quiet, calm ambiance and aesthetics for family meetings.

We have that private space…that overlooks the courtyard. It’s just a lovely quiet area…and people can really relax in there…there’s tea and coffee, and everything about it is a little bit less clinical.

Alternatively, some family members with burning questions and limited time were more concerned with having their questions answered than privacy or aesthetics and did not mind discussing personal issues with the staff in the hallway. “And so even though for us, you’re thinking this is not an appropriate or ideal [location]—for them it is. And that’s what they need at that moment.”

### Ensuring Understanding

The nurses stated they clarified, interpreted, and reinforced the information to patients and family members “because more than likely they won’t have understood it.” This relates to explaining the content and deciphering some doctors’ strong accents. Therefore, it was suggested that it was essential that bad news be delivered in “clear language that is easy to understand.” In addition, nurses suggested it was essential to read people’s body language as this would indicate patients’ and family members’ level of understanding.

[Be] aware that a lot of times people nod and say, “Yes” and then regardless of that, they’re not on board, or that their eyes have glazed over because they can’t believe what they’re being told.

In preparation for a family meeting where wishes were known, some nurses encouraged the patient and family to prepare a list of questions they wanted answered and to speak for themselves during the meeting. After the patient or family had time to digest the news and gather their thoughts, nurses returned to check their understanding. They believed this was important “because they can hear things very differently from what actually happened.”

Nurses stated that reinforcing and explaining the information in various ways through ongoing conversations helped patients and family members absorb the news.

It’s an ongoing conversation and reminding them that “You can ask the same question multiple times. You might change your mind…You might think about it and actually disagree. For whatever reason, we’re here to come back to that conversation as many times as you need.”

## Palliative Care Practice

The second theme centered on meeting patients’ and family members’ physical, psychosocial, and spiritual needs during a pandemic. While many nursing tasks became more convoluted, nurses continued to provide person-centered care to the best of their ability within the constraints imposed upon them.

### Aiming for Holistic Patient Care

One nurse stated that holistic person-centered palliative care included “providing the simple things in life…it’s not just the clinical skills and the assessments, it’s what it looks like, it’s what it feels like.” Patients and family members benefited from holistic care, and nurses felt a sense of satisfaction and personal reward.

I find it very rewarding to be able to give someone peace and reassurance at that time in their life…I guess when it comes to palliative care…everyone expects the care to stop and that nothing can be done. Something can always be done, and to be able to offer that is quite incredible.

***I guess when it comes to palliative care…everyone expects the care to stop and that nothing can be done. Something can always be done, and to be able to offer that is quite incredible.***

In addition, nurses within the PCUs described person-centered care as being culturally and spiritually individualized, incorporating, for example, “possum cloaks…different quilts…[or] turn[ing] the bed to face Mecca, and…[a] spiritual care [person] who can bring [in] different religious items.” Respecting a patient’s individuality allowed nurses to provide care that met the patient’s needs.

We had one patient that brought in eight different-sized pillows [from home]…. Each one had a name, and she liked one in each different spot which made it quite a fun game for the staff and family.…It does make it easier for us to care, it gives us direction.

Family members’ physical and psychosocial needs were met by providing free meals during their visit. This simple expression of care enabled the family to continue spending quality time with the patient. “I often say to them,‘Enjoy your time together as this can…be a really special time…Make the most of it.’”

### Palliative Care Training

One nurse reported that end-of-life care was not prioritized within the regional acute wards but delegated to the junior graduate. Instead, the palliative care patient should be allocated “to the most experienced nurse because the most important person there [is] going to die once.” Therefore, generalist nurses need education and training to support them as they develop experience because “the family dynamics that are involved can be very confronting, especially for junior staff that have very limited exposures.”

Many generalist nurses were reported to have developed skills “on the acuity…chemo competencies and the CVAD, [but] they haven’t had any focus on the way that they communicate.” The nurse continued to explain that symptom management and things like that are quite straightforward, but communication…[as well as] empathy and understanding…is a skill that we need to continually learn and develop…I really feel like it’s the one thing we don’t educate and train enough of.”

***symptom management and things like that are quite straightforward, but communication…[as well as] empathy and understanding…is a skill that we need to continually learn and develop…I really feel like it’s the one thing we don’t educate and train enough of.***

Training and education can take many forms, including observing and imitating skilled clinicians. One nurse learned her communication skills this way and stated, “I followed my mentors around for many years just picking up phrases” and found satisfaction in passing on her knowledge and upskilling the nurses in her team. On the other hand, another senior nurse commented that without education and mentorship from experienced palliative care nurses, generalist nurses did not know what excellent palliative care looked like.

It’s just they have not been taught, and they have not seen that behavior that they could then mirror; they haven’t seen it from their senior nurses. They think you go in, you give some medication, and you leave.

### COVID-19 Impact on Practice

Social distancing measures and visitor restrictions established to prevent the spread of the COVID-19 virus affected nursing practice and communication processes. End-of-life patients were no longer guaranteed a single room because “most of our single rooms…that we would allocate to our palliative care patients…were then taken up with people that were obviously on COVID-19 precautions.” In addition, the format of family meetings had to be flexible “because family aren’t there all the time to get that information, but also they’re not always allowed to come in. So, around COVID-19, there were lots of phone calls.” As well as communication pressures, nurses reported feeling frustrated with visitor restrictions, as they could see the negative impact of social isolation.

I think for me, one of the biggest distresses of COVID-19 is the families who have just been diagnosed with a terminal illness and referred to palliative care and have some time, [they] might even have a year…There’s no one there to give them a hug because they’re not allowed in.

Social restrictions also affected the experience of dying as “people died more happily with all of their family around them if that was normal for them…And now they can only have two in the room at a time.” As a result of acting as gatekeepers, many nurses suffered moral distress and physical and emotional exhaustion.

There’s moral distress to nurses who are having a conversation that is not part of their job…It’s actually not part of our role to restrict loved ones at the end-of-life…and ask them to choose between family members who can be at the bedside.

***There’s moral distress to nurses who are having a conversation that is not part of their job…It’s actually not part of our role to restrict loved ones at the end-of-life…and ask them to choose between family members who can be at the bedside.***

This resulted in workforce shortages due to nurses resigning or being unable to work due to COVID-19 infection. “I just think seeing what COVID-19 has done to our team in the last 12 months is heartbreaking,” but nurses still have not had a reprieve from the demands that have been placed on them.

They are beyond [it]…We’re still calling on nurses for so much more, but their workload hasn’t changed. The rest of the world has sort of gone back to normal outside of hospitals. But the workload…hasn’t changed for them.

## The Environment Matters

Theme three describes the different environments within the wards, the clinical, home-like, and natural, and the effect these have on nurses, patients, and families.

### The Clinical Environment

No dedicated rooms were set aside for palliative or end-of-life patients in the regional hospitals, so they were cared for in acute or subacute clinical environments. While larger rooms were allocated where possible, not all rooms had adequate space for a family member to sleep by the bedside.

A [patient’s] daughter slept on the floor for several days just on a mattress…but that was in a tiny, cramped room, and to get to that side of the bed, we had to walk on her bed—her mattress, on the floor.

Some regional nurses felt that palliative or end-of-life care spaces were more of an afterthought than a priority at their hospitals.

Sometimes…they say, “Oh, this person is taking a little while to die, we’ll just put them in [the other ward] because it’s quieter, and there’s a garden.” Well, no one ever goes out in the garden.

The nurses described the metropolitan PCUs and regional acute wards as clinical, yet the ward could have a warm ambiance and aesthetics if care were taken. “Look, it’s still clinical. It is still within a hospital, but it’s very warm, it’s inviting…We’ve got beautiful artwork throughout the ward [and] I think the layout lends itself to privacy.” In contrast, one nurse felt there was no pride in the appearance of the regional ward at her hospital where end-of-life patients were admitted because “It’s old and dowdy. It has stained carpets which I just find repulsive…and I often think, ‘What would the patients think?’” Another nurse commented that while her hospital was clean and tidy, “It’s stark, it’s clean, and it…smells like a hospital.”

### A Home-Like Environment

According to the nurses’ responses, there was a marked difference between the room layouts and home-like ambiance and aesthetics in the metropolitan PCUs compared to the regional acute wards despite both being regarded as clinical environments. One of the PCU’s home-like spaces was described this way:

We designed the backboard of the beds to be quite home-like; they’re a lovely timber sort of veneer…There’s really very little clinical in the rooms at all, most things we can take in when we need and take out as we need…Every room’s got a day bed with drawers for someone to stay in. They’ve got their own wardrobe…And they can accommodate someone to stay overnight with plenty of space…People can bring all their own things and have flowers and pictures all over the walls. And you don’t feel suffocated.

Donated quilts made by volunteers and a bar fridge, “so if people want to keep drinks or chocolate in there, they could,” were decor that could help create a home-like ambiance. When asked about creating a home-like environment for palliative and end-of-life patients, all nurses had many suggestions for improvements to rooms to “make it feel like home…and not a hospital.” This included decorating walls and shelves with “photos, photo albums, and their flowers or their teddy bears, or any drawings that the grandchildren have done” and creating a space where family members could be self-sufficient.

### Connection to Nature

Most nurses reported that palliative or end-of-life patients’ hospital rooms had views of bushland, gardens, or courtyards. They believed incorporating greenery was essential for well-being and watching outside activities provided interest and a distraction for patients. One of the regional hospitals had a gardened courtyard that staff, patients, and family could easily access. One metropolitan PCU provided direct garden access within some of the patient rooms.

When nurses were asked what they would like to see in their ideal palliative care environment, they commented that gardens were significant in that they helped patients and family members reminisce about their gardens or favorite flowers. One nurse believed that the value of outdoor access was more important for patients themselves rather than for family members because “I think the families are there to see their relative and then are more likely to go off and have a coffee than want to just be in the garden for the sake of it.”

In contrast to the above comment, another nurse suggested that an ideal palliative environment would be located on the “ground floor with doors that open with a breeze that can come through and a proper garden [so] people can go out, and a sitting area where they can sit with family and talk.” The nurse added that when family members were not there, she would love to have a team of trained palliative volunteers who “can come and sit and listen to people or just sit by a bed or rub someone’s hands or push them outside into the air or go and buy them a coffee.”

## Discussion

The findings from this study not only highlight the difference between palliative care provision within dedicated palliative spaces such as PCUs in metropolitan areas and acute wards in regional hospitals regarding ambiance and aesthetics, family meeting spaces, and experienced palliative care nurses but also highlight similarities regarding language use and the impact from the COVID-19 pandemic.

The COVID-19 virus created a nontherapeutic hospital environment for staff, patients, and family members in the metropolitan PCUs and regional acute wards. Where previously, many patients felt stressed within the acute hospital environment due to noise, unfamiliarity, and loss of control (Abuatiq et al., 2020; Miller et al., 2022b), COVID-19 impacted the physical environment as single-patient rooms were reallocated to COVID-19 patients and patient’s psychosocial environment through isolation from their loved ones with numerous research highlighting the resultant suffering and psychological harm (Bloomer & Walshe, 2021; Kirby et al., 2021; Klinger et al., 2022; Rokach, 2022; Usher et al., 2020), human conditions that the palliative approach aims to prevent (WHO, 2020). Nurses forced into a gatekeeping role they knew was harmful to patients and families clashed with their values of providing excellent palliative care. This resulted in moral distress, reducing coping capacity and/or resignation, and numerous studies support these findings (Jackson et al., 2022; Tye, 2022).

Palliative care registered nurses have wide-ranging responsibilities within their scope of practice (Nursing and Midwifery Board of Australia, 2016) to not only assess and administer care tasks but to educate, inform, comfort, and support their patients and family members in order to meet their physical, emotional, social, and spiritual needs (Schroeder & Lorenz, 2018). Empathetic, open, and honest communication by the whole team plays an essential role in achieving this (Stevens et al., 2020). However, the current study found that many nurses and doctors in both the metropolitan PCUs and regional acute wards used “soft” language and metaphors instead of “death” and “dying” words, which according to McLennon et al. (2013) and Collins et al. (2017) had the potential to dilute the gravity of the message, leaving patients and family members confused and unprepared. Further, nurses reported that some doctors avoided conducting bad news conversations, resulting in late referrals to the palliative care team, and denying patients and family members the time to prepare for end-of-life physically, financially, and emotionally (Miller et al., 2022a).

Despite the availability of protocols and guidelines, education, and training programs (Alelwani & Ahmed, 2014; Carrard et al., 2020; Sobczak, 2022; von Blanckenburg et al., 2020), many health professionals were not comfortable with the palliative care aspect of their role (Keeley, 2017; Matthews et al., 2019; Miller et al., 2022a). The need for health professionals to conduct conversations about death and dying early in the disease trajectory, while not new, needs a constant reminder (Miller et al., 2022a). Learning by example, mirroring, and being mentored by senior staff within trusted relationships (Kangas-Niemi et al., 2018) were suggested by participants as a solution for upskilling novice palliative nurses and are supported in the literature (Donnelly & Dickson, 2013). However, it was found that the number of experience palliative staff to provide mentoring and guidance for novice nurses were limited in the regional acute wards.

Participants described a home-like feel as being created by items such as timber bedheads, day beds, storage drawers, hidden clinical equipment, and nature views alongside photos, drawings, and other personalized items. Richards and McLaughlan (2023, p. 4) support these findings and add that the inclusion of timber and how it is used within the patient’s room “makes it look more homely…it’s warmth has comforting tones [which] gives the room a more homelike decor, and a ‘not-too-hospitalish’ naturalness.” All participants commented on the importance of a home-like environment that could still allow clinical care to occur within the room when needed by having equipment that was either hidden behind panels or easily removed, which is supported in the literature (Richards & McLaughlan, 2023).

Green spaces were appreciated, providing a place of respite or restoration for patients, families, and staff. The design of the metropolitan PCUs incorporated quiet rooms with green outlooks where family meetings could be held, whereas this was not available in the regional acute wards. Further, limited family space in some rooms, poor family amenities, limited or nonexistent outdoor access, and a clinical multipurpose room used for family meetings highlight the nurses’ frustration in providing care in their regional hospitals where it seemed palliative, or end-of-life care received little priority and minimal budget allocation. Recent research by McLaughlan et al. (2022) reported that nurses felt frustrated and distressed when unable to provide the care they aspired to within environments that did not support the philosophy of palliative care, which was evident in the current study.

In order to meet the goals as set out in the National Palliative Care Strategy 2018 (Department of Health and Aged Care, 2018), hospital environments for palliative and end-of-life patients and families need to be fit for purpose, with care being provided by health professionals that are well educated and skilled in a palliative approach. While the government has committed to these strategic goals, regional Victorian hospitals need increased government prioritizing and funding to achieve this vision.

## Conclusion

A therapeutic environment for palliative and end-of-life patients requires a home-like physical environment and a psychosocial environment created by staff who can provide holistic palliative care. In regional areas of Victoria, Australia, where there are no dedicated palliative spaces such as PCUs, care is provided by generalist nurses. In addition to education, providing holistic palliative care requires mentoring and mirroring expert practice to increase the expertise and capacity of the palliative care workforce. This research will add to the body of knowledge to inform clinical practice and training around delivering diagnostic/prognostic information and highlight the importance of a therapeutic ward environment that is fit for the purpose of providing excellent palliative care.

## Strengths and Limitations

The study was conducted during the COVID-19 pandemic, confining recruitment to social media advertisements and data collection through online or telephone interviews which may have reduced interview participation. In addition, nurses suffering from pandemic fatigue and limited availability due to shift work may not have had the capacity to participate in the research. Although the sample only consisted of six female registered nurses, the two NUMs within the PCUs, the palliative care NP and the general nurses, provided a rich, in-depth understanding of nurses’ experiences of providing palliative care in regional hospitals in Victoria.

## Implications for Practice

- The ward environment should be fit for the purpose of providing excellent palliative care.
- A home-like environment could be created with the strategic use of warm timbers, adequate space for family, and the covert placement of clinical equipment.
- Junior nurses need mentoring and opportunities to mirror expert practice.
- More education about the normality of death and dying is needed to encourage frank diagnostic/prognostic conversations and earlier referrals to palliative care services.
